# SARS-CoV-2 Neuroinvasion, Inflammatory Neurodegeneration and Alzheimer's Disease

**DOI:** 10.3389/fncel.2022.937961

**Published:** 2022-06-17

**Authors:** Yuhai Zhao, Walter J. Lukiw

**Affiliations:** ^1^LSU Neuroscience Center, Louisiana State University Health Science Center, New Orleans, LA, United States; ^2^Department of Cell Biology and Anatomy, LSU Health Science Center, New Orleans, LA, United States; ^3^Department of Ophthalmology, LSU Health Science Center, New Orleans, LA, United States; ^4^Department of Neurology, Louisiana State University Health Science Center, New Orleans, LA, United States

**Keywords:** angiotensin-converting enzyme-2 receptor (ACE2R), Alzheimer's disease (AD), neurological sequelae of COVID-19, microRNA, AD continuum, SARS-CoV-2 neuroinvasion, post-acute sequelae of SARS-CoV-2 infection, single-stranded RNA (ssvRNA)

## Introduction

COVID-19 is a rapid onset, highly transmissible and lethal viral pneumonia caused by the novel severe acute respiratory syndrome coronavirus SARS-CoV-2. SARS-CoV2 is currently responsible for a serious global pandemic in which about ~520 million people have been infected and ~6.5 million have died (https://www.worldometers.info/coronavirus/coronavirus-death-toll/; last accessed 27 May 2022). As a member of the *Betacoronavirus* genus in the family of enveloped, single-stranded RNA (ssRNA) viruses *Coronaviridae*, SARS-CoV-2 invasion of susceptible human hosts is a complex epidemiological, microbiological, immunological and neurological process. SARS-CoV-2 infection initially requires the interaction of a highly antigenic SARS-CoV-2 viral surface spike (’S1') glycoprotein with the naturally occurring angiotensin-converting enzyme-2 cell surface receptor (ACE2R) of the human host. While ACE2R densities appear to be highest in cholesterol- and sphingolipid-enriched lipid raft domains of multiple epithelial and endothelial cells of the human respiratory tract, this type 1 dipeptidyl carboxydipeptidase trans-membrane protein has been identified on every human host cell type so far analyzed except for erythrocytes (Hill et al., 2021; Palacios-Rápalo et al., 2021; Zhao et al., 2021; Kirtipal et al., 2022; Lukiw et al., 2022). SARS-CoV-2 virus therefore has potential to damage almost every tissue and organ system within the body and to induce a serious multi-organ system failure involving pulmonary, cardiovascular, endocrine, hematologic, renal, gastrointestinal, dermatologic, immunological, psychiatric and/or neurological manifestations. Cholesterol, sphingolipid and other lipid levels in the blood serum and cell membrane appear to modulate viral infectivity, and persons with underlying chronic lipid-associated diseases including cardiovascular disorders, cancer, obesity, chronic lung disease, diabetes or neurological disease have the worst prognosis for COVID-19, and are the most likely to develop acute respiratory distress syndrome and lethal pneumonia (Palacios-Rápalo et al., 2021; Chidambaram et al., 2022; Chiner-Vives et al., 2022). The remarkable and extraordinary capacity of SAR-CoV-2 to attack many different kinds of human host cells simultaneously may help explain the variability in symptoms and overall general feeling of malaise reported by COVID-19 infected patients.

ACE2R presence is also easily and abundantly detected in the majority of cell types of the brain, CNS, neurovasculature, choroid plexus and tracts involving the brain's visual processing systems (Hill et al., 2021; Hixon et al., 2021; Zhao et al., 2021; Lukiw, 2022a; Piras et al., 2022). The highest ACE2R expression found to date in the human CNS has been localized to the neurons of the medulla oblongata and pons in the brainstem, containing the brain's medullary respiratory center. This may in part explain the vulnerability of SARS-CoV-2 infected patients to serious respiratory distress (Zhao et al., 2021; Lukiw et al., 2022; Molina-Molina and Hernández-Argudo, 2022). The normal physiological role of the ACE2R is in the binding and maturation of angiotensin, a circulating peptide hormone derived from its precursor angiotensinogen. Angiotensin functions as a vasoconstrictor and regulates blood flow and blood pressure in the systemic- and neuro-vasculature, the latter an area of the human vascular neurobiology that, besides the limbic system, is targeted in Alzheimer's disease (Carlson and Prusiner, 2021; Xia et al., 2021; Zhao et al., 2021; Lukiw et al., 2022; Sirin et al., 2022; Villa et al., 2022; AD). This ’Opinion paper' will briefly review and comment on our current understanding of SARS-CoV-2 infection of the human CNS and the complex, immediate and long-term contributions of this lethal *Betacoronavirus* to the altered molecular-genetic and pathophysiological mechanisms that characterizes AD-affected brain.

## SARS-CoV-2 Virus—the Causative Agent of COVID-19 and Inflammatory Neurodegeneration

Firstly, it is important to overview the major points of the structure of SARS-CoV-2 and the mechanism of its infectivity. The SARS-CoV-2 virus possesses an extraordinarily large, positive-sense ssRNA genome of about ~29,903 nucleotides [nt; SARS-CoV-2 isolate Wuhan-Hu-1, National Center for Biological Information (NCBI) GenBank Accession No. NC_045512.2; last accessed 27 May 2022; Ke et al., 2020; Sah et al., 2020; Wu et al., 2020; Mousavizadeh and Ghasemi, 2021]. As a *Betacoronavirus*, SARS-CoV-2 is in the same family of other pathogenic human influenza-causing ssvRNA *Coronaviruses* such as hCoV-OC43, HKU1, 229E, severe acute respiratory syndrome (SARS) and Middle East respiratory syndrome coronavirus (MERS-CoV; Sah et al., 2020; Mousavizadeh and Ghasemi, 2021; Raghuvamsi et al., 2021). SARS-CoV-2 consists of a ~100 nm diameter spherical virion particle containing a nucleocapsid core enclosing its ssRNA genome within a compact lipoprotein envelope (Ke et al., 2020). The ssRNA genome of SARS-CoV-2 structurally resembles a “typical” messenger RNA (mRNA) possessing a 5′ methyl cap structure, a 3′ poly(A) tail and ~10–14 overlapping open reading frames (ORFs) with minimal spacer regions, encoding about ~30 proteins, not all of which have been fully characterized (Ke et al., 2020; Sah et al., 2020; Raghuvamsi et al., 2021). SARS-CoV-2 is highly neurotropic toward cells and tissues of the brain, visual system and CNS (Song et al., 2020; Hill et al., 2021; Zhao et al., 2021; Lukiw, 2022a) and orchestrates a highly coordinated and multipronged strategy to impede host protein synthesis including the accelerated degradation of host cytosolic cellular mRNAs, thus facilitating viral takeover of the host mRNA pool in infected cells (Hosseini et al., 2021; Hill and Lukiw, 2022; Lukiw et al., 2022). As a ssRNA virus SARS-CoV-2 is representative of the most common type of emerging viral disease in humans (due to the high mutation rate in RNA compared to DNA viruses) that possess extremely high mutation rates of up to 10^**6**^ times higher than that of their host cells (Pachetti et al., 2020; Finkel et al., 2021). The major structural features of the SARS-CoV-2 virion particle include the envelope (’E'), membrane (“M”), nucleocapsid (’N'), replicase (’R'; an RNA-dependent RNA polymerase) and a surface spike (’S1') protein in addition to several accessory viral-encoded proteins (Ke et al., 2020; Finkel et al., 2021; Siniscalchi et al., 2021). Interestingly the SARS-CoV-2 viral lipoprotein envelope is decorated with ’E', ’M', and/or ’S1' proteins. This ’S1' protein is a class 1 homo-trimeric viral fusion protein possessing distinctive ’head' and ’stalk' domains essential for host cell entry via the ACE2R (see below; Ke et al., 2020; Lukiw, 2021; Raghuvamsi et al., 2021). Interestingly SARS-CoV-2 possesses one of the largest genomes of all known ssRNA neurotropic viruses and a correspondingly large target for potential interaction with natural cellular sncRNA and miRNA (Finkel et al., 2021; Mousavizadeh and Ghasemi, 2021; Hill and Lukiw, 2022).

## Viral and Microbial Infection of the Brain and CNS

**Figure 1:**
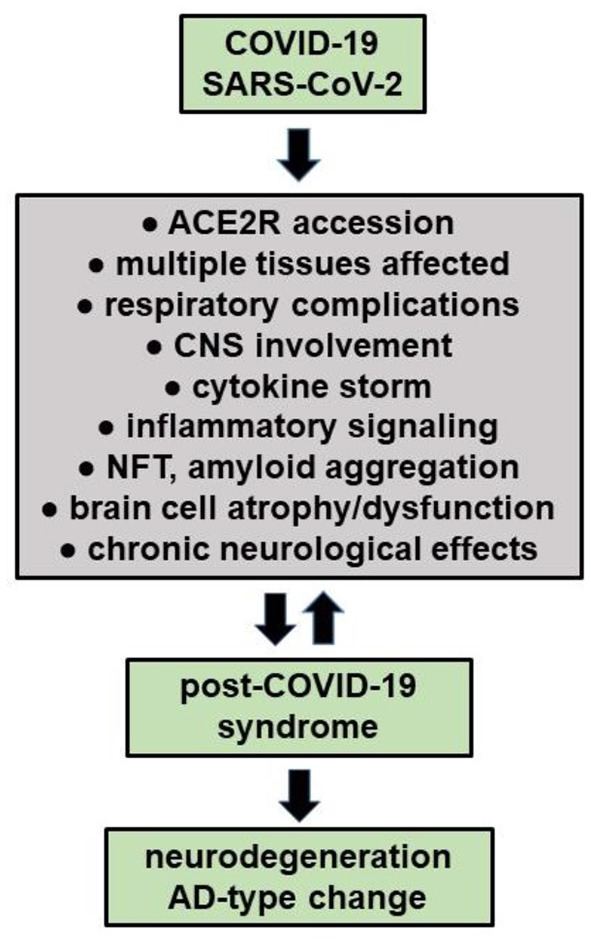
COVID-19 infections may have long-term effects termed “Long COVID' or “post-COVID-19 syndrome”, especially in the elderly and persons with pre-existing disease. It is also evident that persons with underlying chronic disease have the worst prognosis for COVID-19 infection and are the most likely to develop acute respiratory distress syndrome, post-COVID-19-syndrome, a chronic or lethal pneumonia and/or neurological complications (see manuscript text; Palacios-Rápalo et al., 2021; Chidambaram et al., 2022; Chiner-Vives et al., 2022; Visco et al., 2022). Interestingly, a pre-existing diagnosis of AD predicts the highest risk for COVID-19 yet found, driving the highest mortality rate amongst any classification of aged individuals (Nagu et al., 2020; Yu et al., 2021). Recent epidemiological and neurological studies also suggests that persons with COVID-19, and especially severely affected COVID-19 patients, may be predisposed to the development of neurodegenerative disorders that include AD due to latent viral effects on CNS structure, function and homeostasis (Mao et al., 2020; Pacheco-Herrero et al., 2021; Chung et al., 2022; Piekut et al., 2022; Piras et al., 2022; Radhakrishnan and Kandasamy, 2022; Stefanou et al., 2022).

Viral and other microbial infections of the brain and CNS have long been known to contribute, amplify or propagate many of the same neuropathological, inflammatory and neurodegenerative changes as is observed over the entire AD continuum (see below; Lingor et al., 2022; Lukiw et al., 2022; Piekut et al., 2022; Sirin et al., 2022; Szabo et al., 2022). Emerging evidence indicates that both DNA and RNA viruses, such as the human double-stranded DNA (dsDNA) Herpes simplex type 1 and 2 (HSV-1, HSV-2), the human cytomegalovirus (HMCV), the Epstein-Barr virus (EBV), and the ssRNA viruses hepatitis C virus (HCV; *Herpesviridae*), human influenza A viruses (H1N1/H3N2; *Orthomyxoviridae*), Zika virus (ZIKVs; *Flaviviridae*), MERS-CoV (*Coronaviridae*), severe acute respiratory syndrome coronavirus 2 (SARS-CoV-2; *Coronaviridae*) and a remarkably large number of bacteria of the genus *Bacteroides, Borrelia, Chlamydia, Treponema, Porphyromonas, Prevotella, Tannerella, Fusobacterium, Aggregatibacter, Eikenella and Helicobacter*, as well as several other eukaryotic parasites (e.g., *Toxicara; Toxoplasma*) or fungi (*Aspergillus; Candida*) and others have been implicated in the etiopathology of inflammatory neurodegenerative diseases including AD. There is also evidence that a syntrophic consortium of complex microorganisms together, known as biofilms, may be involved and additionally contribute to the neuropathology of AD, however the combination of SARS-CoV-2 invasion with other microbes has not been well studied (Chakravarthi and Joshi, 2021; Piekut et al., 2022; Protto et al., 2022). Importantly, all microbial infections of nervous tissues as described above contribute to the development of a microbial- or viral-induced cytokine storm, a smoldering and progressive inflammatory neurodegeneration and the appearance of neurofibrillary tangles (NFT), amyloid aggregation and related amyloidogenic processes as are observed during the course of AD (Ball et al., 2013; Hosseini et al., 2021; Pogue and Lukiw, 2021; Vigasova et al., 2021; Choe et al., 2022; Lee et al., 2022; Lingor et al., 2022; Piekut et al., 2022; Protto et al., 2022; Sirin et al., 2022; Figure 1). These neuropathological features become more pronounced over the progression of the AD continuum. Importantly strikingly similar neuropathology and biomarkers for amyloidogenesis and inflammatory neurodegeneration have also been observed in stressed human neuronal-glial cells in primary culture and in transgenic murine models of AD (TgAD; Hill et al., 2009; Ball et al., 2013; Vigasova et al., 2021; Choe et al., 2022; Lee et al., 2022; Lingor et al., 2022).

## Alzheimer's Disease (AD), Microbial Invasion and Inflammatory Neurodegeneration

We next briefly review AD in the context of microbial invasion. AD represents a slowly developing, irreversible, progressive, age-related pro-inflammatory neurodegenerative disorder of the human limbic system specifically targeting the human brain neocortex, hippocampal formation and related neuroanatomical regions including the neurovasculature of the CNS (Alzheimer et al., 1995; Lane et al., 2018; Trejo-Lopez et al., 2021). Due in part to the aging population and demographics the global incidence and prevalence of AD is sharply increasing and currently represents the largest cause of behavioral and age-related memory impairment and cognitive decline in industrialized societies (Tahami Monfared et al., 2022; https://alz-journals.onlinelibrary.wiley.com/doi/full/10.1002/alz.12638; https://www.alz.org/media/documents/alzheimers-facts-and-figures.pdf; last accessed 27 May 2022). Three broad phases of AD have been described—sometimes referred to as the *AD continuum*—categorized from pre-clinical AD (also known as “*the prodromal period*”) to mild cognitive impairment (MCI) to mild, moderate and severe AD. This *AD continuum* is further characterized neuropathologically by 7 major changes within the brain: **(i)** by the appearance of hyperphosphorylated tau proteins organized into twisted neurofilament bundles that appear as NFT; **(ii)** by the accumulation of amyloid-beta (Aβ) peptides that aggregate as dense, insoluble lipoprotein deposits called ’amyloid' or ’senile' plaques (SP); **(iii)** by astrogliosis and glial cell proliferation; **(iv)** by alterations in the innate-immune response, increases in inflammatory signaling and the up-regulation of pro-inflammatory cytokine and other biomarkers; **(v)** by progressive cytoskeletal and synaptic disorganization culminating in brain cell atrophy, synaptic signaling disruption, neuronal cell death and progressive neurodegeneration (Alzheimer et al., 1995; Lane et al., 2018; Trejo-Lopez et al., 2021; Zhou et al., 2021; Bethlehem et al., 2022); **(vi)** by weakened gastro-intestinal (GI) tract, neurovascular and blood-brain barriers allowing the influx of viral, microbial and other neurotoxins into brain and CNS compartments; and **(vii)** by deficits in the innate-immune system (see below; Moir et al., 2018; Li et al., 2021; Vigasova et al., 2021; Piekut et al., 2022). The appearance or onset of these 7 major changes typically begins many years prior to when clinical cognitive and behavioral symptoms emerge over a typical AD time course of several decades, and is conducive to an elevated risk of both chronic inflammatory neurodegeneration and opportunistic microbial infection (Lane et al., 2018; Trejo-Lopez et al., 2021; Vigasova et al., 2021; Bethlehem et al., 2022; Choe et al., 2022). The relatively recent discovery that 39-43 amino acid Aβ peptide species have antimicrobial properties further supports the possibility of a contribution of microbial—and in particular a viral-involvement to AD, an infectious etiology to AD, and suggests that the up-regulation of Aβ peptide abundance and SP formation may represent in part a cellular innate-immune response to both viral and/or other microbial infection (Moir et al., 2018; Li et al., 2021; Vigasova et al., 2021).

## SARS-CoV-2, AD and Increased Susceptibility to COVID-19

Multiple epidemiological studies indicate that about ~35% of all COVID-19 patients experience neurological and neuropsychiatric symptoms, and a pre-existing diagnosis of AD predicts the highest risk of COVID-19 infection yet identified, with the highest mortality among elderly AD patients (Song et al., 2020; Zhao et al., 2021; Choe et al., 2022; Chung et al., 2022). Very recent evidence indicates that up to ~45% of all COVID-19 patients develop a mild-to-severe encephalopathy and encephalitis due to complications arising from viral-induced cytokine storm, elevated inflammatory signaling and/or anti-neural autoimmunity, sometimes referred to as a “cytokine storm syndrome” (Mao et al., 2020; Vigasova et al., 2021; Piekut et al., 2022). Just as is consistently observed in AD, the pro-inflammatory cytokines interleukin-1beta (IL-1β), IL-8, IL-1RA and IL-18 and serum neurofilament light (NF-L) chain protein in patient CSF and blood serum, biomarkers for all-cause inflammatory neurodegeneration are significantly associated with COVID-19 severity, and patients with AD appear to be associated with more severe complications of COVID-19 including increased morbidity and mortality (Mao et al., 2020; Krey et al., 2021; Chung et al., 2022; Guasp et al., 2022; Zetterberg and Schott, 2022).

Here we cite 7 recent, highly relevant and independently confirmed examples of clinical, experimental and post-mortem studies: **(i)** SARS-CoV-2 mRNA and multiple SARS-CoV-2 proteins such as the ’S1' spike protein are readily detected in the post-mortem brains of COVID-19 patients, in the brains of experimental COVID-19 murine models, in human brain organoid systems and in cultured neuronal cells infected with SAR S-CoV-2, and their abundance exhibits a positive correlation with ACE2 mRNA levels (Song et al., 2020; Xia et al., 2021; Lingor et al., 2022; Petrovszki et al., 2022; Villa et al., 2022); **(ii)** persons with a diagnosis of AD represent an extremely vulnerable group at high risk of contracting COVID-19 with a tendency to develop more severe symptoms with a more dismal prognosis and higher morbidity, in part because of overlapping risk factors and common pathological and/or pathogenetic mechanisms (Krey et al., 2021; Villa et al., 2022); **(iii)** analysis of primary health records of over 13,300 individuals in the UK that tested positive for COVID-19 indicated that a pre-existing diagnosis of AD predicted the highest risk for COVID-19 yet found, driving the highest mortality rate among any classification of aged individuals (Nagu et al., 2020; Yu et al., 2021); **(iv)** that COVID-19 morbidity and mortality are elevated in AD due to multiple pathological changes in AD patients such as the overexpression of the ACE2R, the cytokine/chemokine storm associated with each disorder, and various ancillary complications of AD including cardiovascular/neurovascular disease, diabetes, delirium, inadequate hygiene and/or other environmental, hormonal and/or lifestyle alterations associated with AD (Hill et al., 2021; Ramos et al., 2021; Xia et al., 2021; Zhao et al., 2021); **(v)** SARS-CoV-2 infection significantly increases neurological, physiological and psychological stress, thus aggravating pre-existing pro-inflammatory reactions such as the viral-induced cytokine storm while supporting the progressive deterioration of neuronal form and function and accelerating the progression of AD (Hu et al., 2021; Pacheco-Herrero et al., 2021); **(vi)** the occurrence of long-lasting neurological symptoms after SARS-CoV-2 infection indicates a prolonged impact on the brain and CNS affecting the same neuroanatomical regions known to be involved in neurodegenerative events as is observed in AD (Song et al., 2020; Krey et al., 2021; Lingor et al., 2022); and **(vii)** importantly, persons infected with COVID-19 exhibit a significant disruption in the abundance, speciation and complexity of host cell microRNA (miRNA) populations and transcriptomic alterations known to be similarly altered in AD brain—some specific examples of which are briefly discussed below (Gordon et al., 2021; Li et al., 2021; Pogue and Lukiw, 2021; Azhar et al., 2022; Kucher et al., 2022; Maranini et al., 2022).

## SARS-CoV-2 Infection and Natural Host microRNAs

Small non-coding RNAs (sncRNAs) known as microRNAs (miRNAs) have emerged as extremely informative diagnostic, prognostic and therapeutic biomarkers in inflammatory and infectious disease including incapacitating viral infections of the brain and AD. For example, increases in the pro-inflammatory NF-kB (p50/p65)-inducible miRNA-146a and miRNA-155, significantly up-regulated in AD- and prion-affected brain and CNS and implicated in pathological disruption of the innate-immune system, altered microglial-regulated waste-product clearance and complement factor H (CFH)-mediated complement activation, most often accompanies the viral-, bacterial- and other microbial-mediated infections of all brain cells and tissues examined to date (Slota and Booth, 2019; Li et al., 2021; Pogue and Lukiw, 2021; Azhar et al., 2022; Choe et al., 2022; Kucher et al., 2022; Maranini et al., 2022; Pogue et al., 2022). Importantly, the abundance, speciation and complexity of miRNA populations varies considerably in the individual human host and because miRNAs can target and inactivate ssRNA viruses such as H1N1/H3N2, Zika virus and SARS-CoV-2 may help to explain individual heterogeneity in the susceptibility to systemic attack and infection by human ssRNA viruses (Azhar et al., 2022; Hill and Lukiw, 2022; Kucher et al., 2022).

It is noteworthy that a broad, non-random spectrum of miRNAs are significantly disrupted in abundance in AD brain and that the SARS-CoV-2 ssRNA genome can specifically recognize and ’sponge' between 857 and 2,654 miRNA-SARS-CoV-2 pairings (there are about 2,654 human miRNAs so far identified; Pierce et al., 2020; Siniscalchi et al., 2021). These actions alone may modulate both natural miRNA abundance, function and the invasiveness potential of SARS-CoV-2 in neural tissues in the brain, visual system and CNS in AD-affected brain. Often overlooked is that the large size of the SARS-CoV-2 ssRNA genome at 29,903 nt, the enormous number of SARS-CoV-2 particles involved in a typical infection and large number of miRNA binding sites within its sequence may also act as a “sponge” to bind specific free miRNAs. These intrinsic SARCoV-2 parameters would therefore down-regulate, deplete and disrupt the abundance and natural levels of free miRNAs within the cell creating metabolic and signaling instability in brain cells while supporting neuro-inflammation and AD-type change (SARS-CoV-2 isolate Wuhan-Hu-1, National Center for Biological Information (NCBI) GenBank Accession No. NC_045512.2; last accessed 27 May 2022; Ke et al., 2020; Sah et al., 2020; Wu et al., 2020; Mousavizadeh and Ghasemi, 2021; Hill and Lukiw, 2022).

## SARS-CoV-2 - The Short- and Long-Term Neurological Sequelae

Hours-to-days after SARS-CoV-2 infection into susceptible *Homo sapiens* there typically results in a persistent cough, shortness of breath, fever, viral sepsis, hypoxemic respiratory failure and rapid onset viral pneumonia. SARS-CoV-2 invasion also causes overall worsening of underlying and existing chronic cardiac, respiratory and other pathological disorders that include atrial fibrillation, asthma, bronchitis, chronic obstructive pulmonary disease (COPD), cystic fibrosis/bronchiectasis, emphysema, interstitial lung disease, pleural effusion, pulmonary fibrosis, lung cancer, pre-existing pneumonia, metabolic syndrome and venous thromboembolic diseases (alphabetically ordered; https://www.unitypoint.org/homecare/article.aspx?id=2448b930-1451-43e4-8634-c0c16707c749; last accessed 27 May 2022; Kallet et al., 2019; Lee et al., 2021; Zuin et al., 2021; Stefanou et al., 2022; Visco et al., 2022). It has been observed that about ~75% of hospitalized COVID-19 patients have at least one COVID-19-associated comorbidity and COVID-19 patients with underlying chronic illnesses are more likely be affected with a more adverse and unfavorable prognosis (Chiner-Vives et al., 2022; Crivelli et al., 2022; Kirtipal et al., 2022).

Early in the COVID-19 pandemic it was also noted that many patients, during or after COVID-19 infection over the “short term” complain of a general malaise and extra-respiratory neurological symptoms including confusion, delirium, headache, mental and psychiatric disorders, disorders in mood (depression and dysthymic disorder), disturbances in sleep (insomnia), cognitive and memory impairment, “brain fog”, deficiency in smell (anosmia) or taste (ageusia), muscle weakness and myalgia, sensorimotor deficits, dysautonomia as well as convulsions and/or peripheral neuropathies that include Bell's palsy and peripheral neuropathies with pain (Gupta and Jawanda, 2022; Lingor et al., 2022; Stefanou et al., 2022). COVID-19 associated ocular manifestations have been also documented to include a wide range of ophthalmic symptoms associated with eye irritation (chemosis), conjunctivitis, conjunctival hyperemia, anterior uveitis, retinitis, and optic neuritis and in advanced COVID-19 infection with visual and perception disturbances including visual disorientation and hallucinations, especially in elderly COVID-19 patients (Hill et al., 2021; Hixon et al., 2021; Lin et al., 2021; Reinhold et al., 2021; Al-Namaeh, 2022; Lukiw, 2022a,b). Accumulating evidence indicates an especially high prevalence of prolonged neurological symptoms among COVID-19 survivors and most of these afflictions and neurological disruptions persist as the long-term neurological sequelae of COVID-19 also known as “long COVID” or “post-COVID-19 syndrome” (Nepal et al., 2020; Song et al., 2020; Ahmed et al., 2022; Sanyaolu et al., 2022; Visco et al., 2022; Figure 1).

## Discussion and Summary

Since the first cases of SARS-CoV-2 infection and COVID-19 disease were reported in December 2019 (2019-nCoV; Chen and Yu, 2020) the full spectrum of neurological sequelae to SARS-CoV-2 viral invasion is beginning to emerge. COVID-19 disease ranges from asymptomatic or mild cases up to severe life-threatening complications and a highly lethal pneumonia. “Long COVID” or “post-COVID-19 syndrome” is emerging as a complex long-term disorder with extended and heterogeneous symptoms in both systemic human physiology and in neurological complications for each individual COVID-19 patient. It is our opinion that the remarkable ubiquity of the ACE2R, the primary receptor for the SARS-CoV-2 virus on multiple cell membrane types of the human host, is probably the reason for the widespread systemic involvement of SARS-CoV-2 invasion, and enrichment of the ACE2R in the limbic regions of the human brain in AD patients is probably why AD patients suffer from an increased incidence and susceptibility to COVID-19 infection (Kallet et al., 2019; Ahmad and Rathore, 2020; Magusali et al., 2021; Sun et al., 2021; Lukiw et al., 2022; Stefanou et al., 2022; Visco et al., 2022). Like many neurotropic viruses with RNA genomes, SARS-CoV-2 has a remarkably broad neuroinvasive capacity and neurons appear to be directly targeted by a particularly virulent infection (Song et al., 2020; Choe et al., 2022; Lukiw et al., 2022). Long-lasting neurological consequences after SARS-CoV-2 infection negatively impacts the brain and CNS in anatomical regions known to be targeted by neurodegenerative events, as is observed throughout all phases of the AD continuum (Lingor et al., 2022; Lukiw et al., 2022; Piekut et al., 2022; Sirin et al., 2022; Szabo et al., 2022). Pre-existing neurological conditions and pathological interactions among the brain, central and peripheral nervous systems (CNS, PNS) and respiratory, cardiovascular and endocrine systems further modulate and/or impact the severity and long-term sequelae of the post-COVID-19 syndrome period (Kallet et al., 2019; Horn et al., 2021; Zuin et al., 2021; Molina-Molina and Hernández-Argudo, 2022; Sanyaolu et al., 2022; Stefanou et al., 2022; Visco et al., 2022). Our recent appreciation that many intractable and invariably fatal neurodegenerative disorders including AD that involve protein misfolding, aggregation and spread are prion disorders provides another dimension for the invasion of SARS-CoV-2, however the interaction between viral structure and infectivity and changes in protein conformation and aggregation are not well understood (Carlson and Prusiner, 2021; Lukiw, 2022b). As both COVID-19 and AD are complex syndromes with a protracted, heterogeneous etiology and progressive neurological involvement, symptomatic patients who experience post-COVID-19 neurological sequelae would clearly benefit from careful clinical monitoring, precision medicine and personalized treatment to better deal with each individual case to optimize the best possible clinical and long-term neurological outcome.

## Author Contributions

YZ and WL collected, analyzed, summarized the literature and formulated an overall opinion on this contemporary topic. WL wrote the article. Both authors contributed to the article and approved the submitted version.

## Author Disclaimer

The content of this manuscript is solely the responsibility of the authors and does not necessarily represent the official views of the NIH.

## Conflict of Interest

The authors declare that the research was conducted in the absence of any commercial or financial relationships that could be construed as a potential conflict of interest.

## Publisher's Note

All claims expressed in this article are solely those of the authors and do not necessarily represent those of their affiliated organizations, or those of the publisher, the editors and the reviewers. Any product that may be evaluated in this article, or claim that may be made by its manufacturer, is not guaranteed or endorsed by the publisher.
